# Supplementary figures and images for: Application of the Luminescence Syncytium Induction Assay to Identify Chemical Compounds That Inhibit Bovine Leukemia Virus Replication
Source: Viruses. 2022 Dec 20;15(1):4. doi: 10.3390/v15010004 (PMC9861517; doi:10.3390/v15010004)

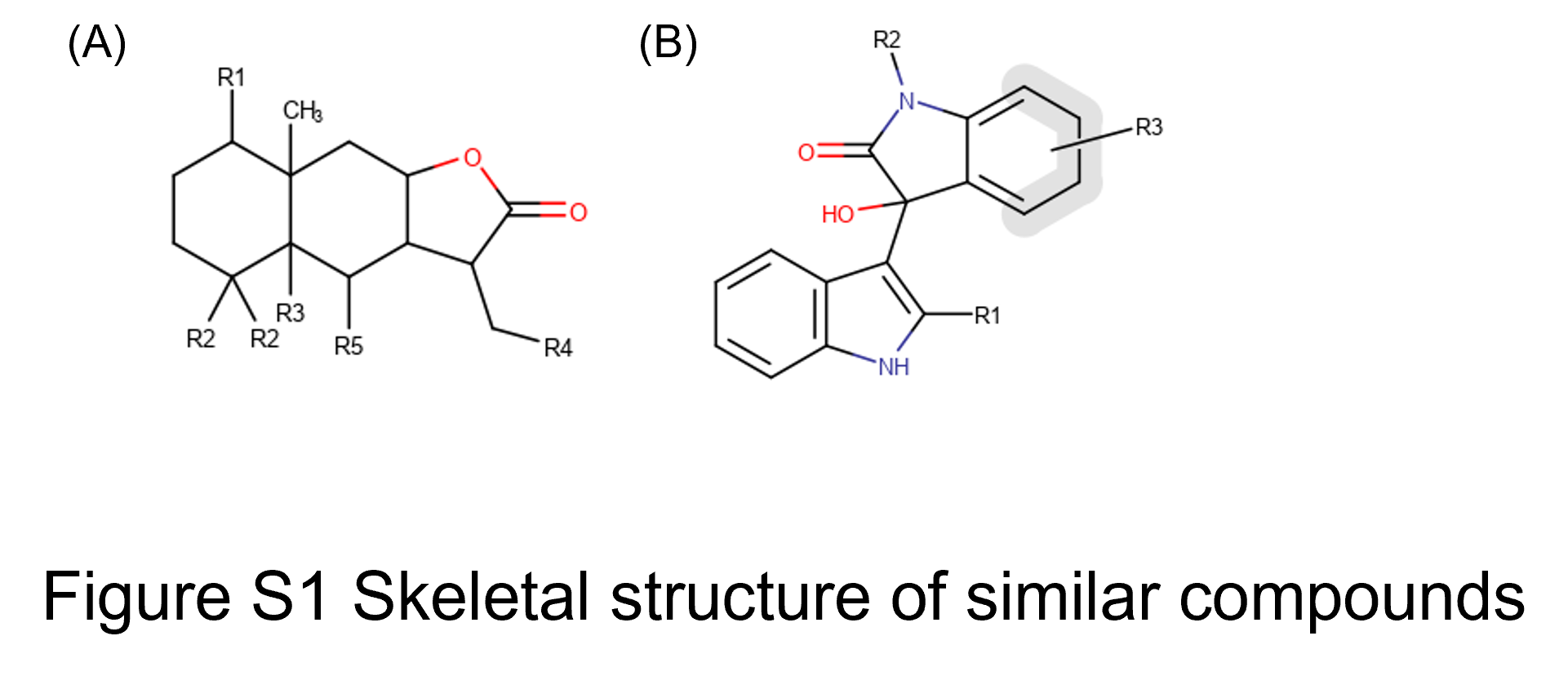

Supplement: Supplementary file 1 [file viruses-15-00004-s001.zip › figure S1.tif]
